# EHMTI-0257. Intractable refractory unilateral hemicrania with autonomic symptoms – a case study of an undiagnosed pontine cavernous malformation

**DOI:** 10.1186/1129-2377-15-S1-D62

**Published:** 2014-09-18

**Authors:** SS Talibi, S Chaal, K Kioulachidis, B Davies

**Affiliations:** 1 Department of Neurology, University Hospital North Staffordshire, Stoke-on-Trent, UK

## 

Cerebral Cavernous malformations (CCM) occur in less than 1% of the general population, and one-third may have more than one lesion. Headaches alone may accompany CCM in up to 10% patients although micro-haemorrhage with accompanying headache is more common.

A 67-year old male presented with a 15-year history of episodes of sudden onset left facial pain with dysesthesia in the fifth cranial nerve V1 distribution and left sided headache. The frequency of facial pain has increased with daily episodes of occurrence, exacerbated by movements. His headache impact score HIT-6 was 71. The patient’s family history is a sister died of a stroke, another from multiple sclerosis and a nephew died at 15-years-old of cerebral haemorrhage. On examination there were signs consistent with a brainstem lesion. Extensive scanning demonstrated no haemorrhage into any lesion and confirming multiple foci including a pontine lesion. Genetic testing confirmed a CCM2 mutation heterozygous deletion of two-nucleotides and 50% risk of inheritance of the c76_77del to family members.

Pontine CCM can precipitate in migrainous-type headache and atypical facial nerve distribution symptoms due to disruption of the trigeminovascular system (TGVS). The Periaqueductal Grey area (PAG) is involved in modulating pain through both ascending and descending projections, the latters connections to the TGVS that inhibits nociceptive afferent information. CCM lesion is also disrupting the Nucleus Raphe Magnus (NRM), acting as a migraine generator.

The diagnosis for the cause of this intractable facial pain and headache is the large pontine CCM lesion disinhibiting fibres of the TGVS.

No conflict of interest.

